# Outcomes of Hospitalized Octogenarians with *E. coli* Bacteremia—Retrospective Cohort Study

**DOI:** 10.3390/pathogens14111154

**Published:** 2025-11-12

**Authors:** Alaa Atamna, Bayan Mahajneh, Yaara Wazana, Shahd Dahamsheh, Haim Ben-Zvi, Jihad Bishara

**Affiliations:** 1 Infectious Diseases Unit, Rabin Medical Center, Beilinson Hospital, Petah-Tikva 49100, Israel; 2 Faculty of Medical and Health Sciences, Tel Aviv University, Tel Aviv 69978, Israel; bayanmahajne@mail.tau.ac.il (B.M.);; 3 Rabin Medical Center, Beilinson Hospital, Petah-Tikva 49100, Israel; 4 Clinical Microbiology Laboratory, Rabin Medical Center, Beilinson Hospital, Petah-Tikva 49100, Israel

**Keywords:** *E. coli* bacteremia, older adults, octogenarians, outcomes

## Abstract

**Background:** *Escherichia coli* (*E. coli*) bacteremia is a significant cause of mortality, particularly in older adults. Limited data exists on clinical outcomes in octogenarians. This study aims to evaluate the clinical outcomes of *E. coli* bacteremia in octogenarians and determine whether appropriate empirical therapy leads to improved outcomes in this specific population. **Methods:** We conducted a retrospective cohort study of hospitalized patients with *E. coli* bacteremia at Beilinson Hospital from January 2012 to December 2022. Clinical characteristics, bacteremia sources, antibiotic resistance patterns, and patient outcomes were analyzed. The primary outcome was 30-day mortality. Multivariate regression was used to assess the impact of empirical antibiotic appropriateness on mortality. **Results:** The study included 2717 patients, of which 1042 (38%) were 80 years or older. Older patients had more comorbidities with increased rates of ischemic heart disease (20% vs. 14%, *p* < 0.01) and congestive heart failure (19% vs. 9%, *p* < 0.01). Patients with 3rd generation cephalosporin resistant strains were more likely to receive inappropriate empiric antibiotic therapy (54% vs. 23%, *p* < 0.01). Although appropriate empirical therapy was associated with improved survival in univariate analysis (19% vs. 28%, *p* < 0.01), it was not an independent predictor of 30-day mortality in multivariate analysis [adjusted OR = 1.10, 95% CI (0.64–1.81), *p* = 0.7]. A lower SOFA score [adjusted OR = 0.17, CI95% (0.01–0.31), *p* < 0.01] was associated with decreased 30-day mortality. Hypoalbuminemia was significantly associated with increased 30-day mortality [adjusted OR = 2.49, CI95% (0.1.56–3.97), *p* < 0.01]. **Conclusions:** *E. coli* bacteremia in octogenarians is associated with significant mortality. While timely appropriate antibiotic therapy is crucial, mortality appears to be more influenced by overall health status, comorbidities, and infection severity. Future research should focus on addressing these factors and developing personalized care strategies to improve survival in this high-risk group.

## 1. Introduction

As the population ages, infectious diseases have emerged as a serious threat causing significant morbidity and mortality in older patients. The incidence of bacteremia increases significantly with age and is associated with poor prognosis [1].

Recent studies estimate that the 30-day mortality rate in *Escherichia coli* bacteremia ranges from 9.6% to 18.2% [2,3]. In a large retrospective study from England that included 180,320 patients with *E. coli* bacteremia; half the patients (51%) were 75 years or older, highlighting the high prevalence of *E. coli* bacteremia in this age group [4].

The mortality rate in patients with *E. coli* bacteremia also increases markedly as the age advances. In a retrospective study that included patients with *E. coli* bacteremia, the overall mortality by age group was (61/136, 16.4%) in old patients and (34/136, 12%) in very old patients (≥80 years) as compared with the younger ones (41/136, 10.4%) [5].

The heightened vulnerability of elderly patients to infections may be attributed to the altered immune response related to aging (immunosenescece) and the loss of physiological reserve that is related to frailty, in addition to comorbidities such as diabetes mellitus, chronic kidney disease, and malnutrition that may impair the ability to mount effective immune responses and lead to an increased vulnerability to infections [6,7].

Early appropriate antibiotic treatment was shown to reduce the odds of mortality in patients with Gram-negative bacteremia. Paul et al. showed in their meta-analysis study that inappropriate treatment was associated with a significant increase in all-cause mortality OR = 1.6 (CI95% 1.37–1.86) [7]. Next, a case–control study on 288 older patients (≥65 years) reported that inappropriate empiric treatment was found to be an independent risk factors of 28-day mortality with an odds ratio of 3.65; *p* = 0.049 [8]. Finally, a recent large retrospective study of 20,000 patients with bacteremia demonstrated that receiving discordant empiric antibiotics was associated with increased mortality with an adjusted odds ratio of 1.46 (95%CI, 1.28–1.66]; *p* < 0·0001) [9].

Data is scarce regarding the impact of appropriate early antibiotic treatment on octogenarians with *E. coli* bacteremia.

This study aims to evaluate the clinical outcomes of *E. coli* bacteremia in octogenarians and determine whether appropriate empirical therapy leads to improved outcomes in this specific population.

## 2. Methods

This retrospective cohort study included all consecutive patients of age ≥18 years with monobacterial *E. coli* bacteremia who were detected during hospitalization or admitted with bacteremia in Beilenson hospital between January 2012 and December 2022. The study cohort was octogenarians (age ≥ 80 years) that were divided into two groups according to the appropriateness of the empiric antibiotics they received: the inappropriate group and the appropriate group. The primary outcome was 30-day mortality. A multivariate regression model was used to assess the impact of empirical antibiotic appropriateness on 30-day mortality.

### 2.1. Definition

*E. coli* bacteremia was defined as at least one positive blood culture of *E. coli* with the onset of bacteremia determined by the timing of blood culture collection (day 0).

Bacteremia source was determined through the isolation of *E. coli* from the bloodstream and also from the respective sites (urine, body fluids from biliary or abdominal cavity, and wounds), or according to the presence of clinical infection symptoms from the respective sites.

Appropriate empirical antibiotic treatment: Whenever in vitro active antibiotics against the *E. coli* isolate were administered within 24 h of culture collection.

### 2.2. Microbiological Methods

Blood was inoculated into the Bactec blood culture aerobic and anaerobic bottles (BD, Franklin Lakes, NJ, USA). Upon receipt in the clinical microbiology laboratory, all Bactec blood culture bottles were incubated in the Bactec FX incubator (BD, Franklin Lakes, NJ, USA) for up to 5 days. Once positive, blood culture bottles were aseptically inoculated into blood agar, MacConkey, and chocolate agar (HyLabs Rehovot, Rehovot, Israel) and incubated for 18 to 24 h at 37 °C and 5% CO_2_. A Gram stain slide was also prepared and reported within 1 h of positivity. Blood cultures with Gram-negative organisms were tested by matrix-assisted laser desorption ionization–time of flight mass spectrometry (MALDI-TOF MS), either from blood culture bottle or, in cases when there was early growth after a couple of hours, directly from early growth colonies.

After 24 h of incubation of the positive blood culture plate, all suspected colonies were noted and all were identified by matrix-assisted laser desorption ionization–time of flight mass spectrometry (MALDI-TOF MS) (Bruker Daltonics, Billerica, MA, USA).

Antimicrobial susceptibility was interpreted according to the guidelines of the Clinical and Laboratory Standard Institute (CLSI, M100) [10] using the disk diffusion method (Oxoid, Basingstoke, UK) or gradient test, Etest (bioMérieux, Marcy l’Etoile, Lyon, France).

### 2.3. Data Collection

Data was retrieved from patients’ medical records in Beilinson Hospital including demographics [age, gender, age adjusted Charlson comorbidity score, residency before admission, previous hospitalization within 3 months of the index hospitalization, prior surgery within 30 days prior the index hospitalization]. In addition, data regarding comorbidities was also retrieved including diabetes mellitus, ischemic heart disease, congestive heart failure, prior stroke, COPD, dementia, chronic kidney failure, hemodialysis, chronic liver disease, solid tumors and hematologic malignancies, organ transplantations, and chronic steroids. Bacteremia related information was documented including the resistance phenotype (3rd generation cephalosporin (3GC) susceptible, 3rd GC resistant *E. coli*, and carbapenem resistant Enterobacteriaceae—CRE), the source of bacteremia, the appropriateness of the empiric antibiotics (see definition), the Sequential Organ Failure Assessment (SOFA) score at bacteremia identification, laboratory markers at bacteremia identification (albumin and creatinine), and finally mortality at different time frames (7, 14, 30 days).

### 2.4. Statistical Analysis

We compared the study variables based on the appropriateness of the empirical antibiotic therapy (appropriate empirical treatment—study group and inappropriate empirical treatment—control group) using bivariate and multivariable logistic regression models. Continuous variables were assessed using the *t*-test when normally distributed and the Mann–Whitney test when they were not normally distributed. Variables with multiple categories were assessed using the Chi-square test or Fisher’s exact test. Variables were included in the multivariable model based on the bivariate analysis if the significance level was <0.01. The odds ratios (ORs) and 95% confidence intervals (CI) were obtained from the logistic regression models. For all analyses, *p* < 0.05 was considered statistically significant. Data analysis was conducted using SAS 9.4 software (SAS Institute Inc.).

The study was compliant with the Declaration of Helsinki and was approved by the Rabin medical center ethics committee with the number of approval 0643-22-RMC. Informed consent was waived because of the retrospective nature of the study.

## 3. Results

### 3.1. Baseline Characteristics of Hospitalized Patients with E. coli Bacteremia

The study cohort included 2717 hospitalized patients with *E. coli* bacteremia, of which, 1042 (38%) were 80 years and older, and 1675 (62%) were below 80 years. Baseline characteristics of hospitalized patients with *E. coli* bacteremia are presented in Table 1. Overall, octogenarians had higher Charlson comorbidity index (median 5 vs. 4, *p* < 0.01) and they arrived less to the hospital from home (61% vs. 67%, *p* < 0.01) and were less hospitalized in the previous 3 months (29% vs. 40%, *p* < 0.01). More octogenarians were admitted to the internal medicine wards compared to non-octogenarians (88% vs. 81%, *p* < 0.01).

### 3.2. E. coli Bacteremia Characteristics in Octogenarians

A proportion of 68% of *E. coli* bacteremia in octogenarians was from three GC susceptible isolates (68% vs. 73%, *p* < 0.01). The source of bacteremia was most frequently of urinary tract origin (61% vs. 51%, *p* < 0.01).

At time of bacteremia presentation, the median SOFA score was 4.5 in octogenarians vs. 4 in younger patients, *p* < 0.01. The median length of hospital stay was 8 days in both groups, *p* = 0.03.

Almost all patients had received early empiric antibiotics (91% vs. 88%, *p* = 0.014), of which, approximately two thirds of both groups had received appropriate empiric therapy (66% vs. 69%, *p* < 0.01) (Table 2).

### 3.3. Impact of the Appropriateness of Early Empiric Antibiotic Treatment in Octogenarians

Among 947 octogenarians who received empiric antibiotic therapy, 691 (73%) received early appropriate empiric treatment, while 256 (27%) received inappropriate therapy (Table 3). The antibiotic types administered in the appropriate therapy group are presented in Table 4. Cephalosporins comprised 51.6% of the appropriate empiric antibiotics followed by aminoglycosides (33.7%). In patients with 3rd GC resistant *E. coli* bacteremia, most patients received aminoglycosides (71%), followed by carbapenems (17.3%) and piperacillin-tazobactam (11.8%).

Patients who were admitted to internal medicine wards were more likely to receive early appropriate antibiotic treatment (91% vs. 77%, *p* < 0.01).

The source of bacteremia significantly differed between groups, with urinary tract infections being more frequent in the appropriate therapy group (65% vs. 49%, *p* < 0.01). In contrast, patients in the inappropriate therapy group had a higher proportion of hepatobiliary (24% vs. 15%, *p* < 0.01) and intra-abdominal infections (13% vs. 5%, *p* < 0.01). Regarding antimicrobial resistance, 3rd GC resistant *E. coli* isolates were twice as prevalent in the inappropriate therapy group (54% vs. 23%, *p* < 0.01).

Patients who received appropriate early treatment had shorter hospital days (8 days vs. 9, *p* ≤ 0.01).

### 3.4. Mortality in Octogenarians with E. coli Bacteremia

Among the 1042 octogenarians with *E. coli* bacteremia, 22% died within 30 days of admission. Univariate analysis of risk factors for 30-day mortality is presented in (Table 5).

Patients who died were more likely to have a higher Charlson score and higher SOFA scores at bacteremia presentation (6 vs. 5, *p* < 0.01, and 7 vs. 4, *p* < 0.01, respectively). They were also more likely to have a previous hospitalization within 3 months (40% vs. 26%, *p* < 0.01) and they required mechanical ventilation more frequently (15% vs. 3.7%, *p* < 0.01). Patients who died were more likely to have had hypoalbuminemia, with 41% having had albumin levels < 3 mg/dL compared to 20% of patients who survived. They also had higher median creatinine levels at bacteremia presentation (1.7 vs. 1.2 mg/dL, *p* < 0.01).

The source of bacteremia significantly differed between groups, with urinary tract infections being more frequent among survivors (63% vs. 53%, *p* = 0.02). In contrast, 3rd GC resistant *E. coli* isolates were more prevalent among those who died (46% vs. 28%, *p* < 0.01).

Patients who received appropriate empiric therapy had a lower 30-day mortality rate compared to those who did not (55% vs. 70%, *p* < 0.01). However, in the multivariate model for risk factors of 30-day mortality (Table 6), early appropriate empiric antibiotic therapy was not significantly associated with improved survival [OR = 1.10, CI95% (0.64–1.81); *p* = 0.7]. Lower SOFA scores at bacteremia presentation were significantly associated with lower 30-day mortality [OR = 0.17, CI95% (0.01–0.31); *p* = 0.7] (Table 6). Finally, hypoalbuminemia was associated with significantly increased 30-day mortality [OR = 2.49, CI95% (1.56–3.97); *p* < 0.01].

## 4. Discussion

Managing *E. coli* bacteremia in octogenarian patients (≥80 years) presents significant challenges due to higher comorbidity burdens, greater disease severity, and worse clinical outcomes. Our findings confirm that octogenarians experience significantly higher mortality rates compared to younger individuals [4]. However, contrary to some existing literature [7,8,9,11], appropriate empiric antibiotic therapy was not independently associated with improved survival in this population. There are several plausible explanations for these discordant results. First, our study cohort is older (≥80 years) compared with the other study cohorts. Indeed, we were not aware of similar studies that included older adults aged 80 years and above with *E. coli* bacteremia. Other factors that accompany advanced age such as comorbidities, immunocompetence, and frailty may outweigh the benefits of timely antibiotics. In our study, higher Charlson comorbidity index scores and SOFA scores were strongly associated with mortality, emphasizing the role of underlying health status and organ dysfunction in determining outcomes. These findings align with studies like Paul et al., which suggest that in older adults with multiple comorbidities, the benefit of appropriate antibiotics may be limited by overall health status [7]. In addition, for a retrospective study that included geriatric patients with sepsis and septic shock in the medical ICU (*n* = 254), the SOFA score was the only independent risk factor for mortality in the geriatric patient population with sepsis [OR = 1.886 95%CI (1.41–2.51), *p* < 0.001] [12].

In our study, we found that hypoalbuminemia was strongly associated with mortality. This finding is in line with other studies. In a prospective study of 434 inpatients, 90 years or older, hypoalbuminemia (<3 g/dL) was associated with increased 1-year mortality [HR = 2.70, 95%CI (1.69–4.32)] [13]. 

Indeed, in our study, patients who received inappropriate empiric therapy were more likely to require mechanical ventilation, reflecting their baseline disease severity rather than the direct effect of antibiotic choice. In addition, patients with 3rd GC resistant *E. coli* bacteremia were less likely to receive “concordant” empiric antibiotics with in vitro activity against the *E. coli* isolate.

Although a multivariate adjustment was conducted, confounding by indication might persist as sicker patients may have been more likely to receive inappropriate therapy due to their clinical instability.

In our study we found no mortality difference in octogenarians with 3rd GC resistant *E. coli* bacteremia compared to those with 3rd GC susceptible *E. coli* bacteremia. This result is in line with a recent study that found that there was no statistically significant difference in 30-day mortality in ESBL-producing *E. coli* compared to non-ESBL *E. coli* BSI [adjusted OR= 1.6 (CI95% 0.7–3.7; *p* = 0.25), despite a delay (>24 h) in the administration of an effective antibiotic in the non-ESBL *E. coli* [14]. This suggests that some deaths might not be preventable even with effective antibiotic treatment particularly in the older population.

The source of bacteremia also played a critical role in patient outcomes. Survivors were more likely to have urinary tract infections, while those who died and those receiving inappropriate empiric therapy often had non-urinary sources such as hepatobiliary or intra-abdominal infections. This is consistent with prior publications [15,16] showing that urosepsis typically responds well to early treatment, whereas more complex infections require aggressive source control interventions. These findings underscore the importance of rapid identification of infection sources and tailored interventions beyond antibiotics.

Our results contrast with studies like Leibovici-Weissman et al., which emphasized that appropriate empiric therapy is a modifiable factor that significantly impacts outcomes in older adults with community-onset bacteremia. This discrepancy may reflect differences in patient populations, as our cohort included predominantly hospitalized octogenarians with higher baseline comorbidities and more severe infections [17].

Given the limited survival benefit of appropriate empiric antibiotics and the strong prognostic role of comorbidities and infection severity, future management must adopt a holistic approach including prioritizing early and aggressive source control, especially for non-urinary infections, alongside comprehensive organ support driven by severity assessment (SOFA score). Furthermore, the high burden of comorbidities necessitates the integration of a rapid geriatric assessment to guide a personalized care approach that allows for tailoring interventions and facilitating early palliative care engagement and advanced care planning.

The study has key strengths mainly with its large sample size of a special population of octogenarians with *E. coli* bacteremia with comprehensive clinical, microbiologic, and outcome data.

This study has several limitations. Its retrospective design limits the ability to establish causality between empiric therapy and clinical outcomes. Selection bias is possible, as the study was conducted at a single center, potentially affecting generalizability. While we adjusted for major confounders, unmeasured factors such as end-of-life care decisions and frailty assessments may have influenced mortality outcomes. Indeed, in our study cohort, a formal frailty assessment was not performed and given the retrospective nature of the study and the lack of important frailty retrievable measures such as the assessment of muscle strength by hand grip measurements, it was difficult to extrapolate a frailty score. Another limitation of the study is that the definition of “appropriate empirical therapy” in the study is limited to in vitro activity and administration of the antibiotic within 24 h. Other pharmacokinetic/pharmacodynamic (PK/PD) parameters such as dose adequacy, route of administration, and tissue penetration was not a part of the definition of the appropriateness of antibiotics. This limitation may have a significant influence on the observed lack of association between appropriate empirical therapy and mortality in the multivariate model.

In conclusion, our study highlights the need for a holistic approach to managing *E. coli* bacteremia in octogenarians. The appropriateness of the empirical antibiotic therapy carried no survival benefit among individuals at older ages, and their impact on mortality is influenced by broader factors such as comorbidities and infection severity. Future research should focus on addressing these factors and developing personalized care strategies to improve survival and quality of life in this high-risk group. In addition, further research is required to assess sub-populations of octogenarians who may benefit from early antibiotic coverage.

## Figures and Tables

**Table 1:** Baseline characteristics of hospitalized patients with *E. coli* bacteremia.

Variable	≥80 Years (*n* = 1042)	<80 Years (*n* = 1675)	*p* Value
Age, median (IQR)	87 (83–91)	67 (57–73)	-
Female gender, *n* (%)	629 (60%)	849 (51%)	<0.01
Age adjusted CCI, median (IQR)	5 (4–8)	4 (2–8)	<0.01
BMI, median (IQR)	26 (23–29)	26(22–30)	0.4
Home residency, *n* (%)	635 (61%)	1124 (67%)	<0.01
Prior hospitalization in 90 days, *n* (%)	306 (29%)	667 (40%)	<0.001
Prior surgery (30 days), *n* (%)	18 (2%)	78 (5%)	<0.001
Admission department, *n* (%)			
Internal medicine	914 (88%)	1364 (81%)	<0.01
Surgery	123 (12%)	283 (17%)	<0.01
ICU	3 (0.3%)	21 (1%)	<0.01
Comorbidities			
Diabetes mellitus, *n* (%)	232 (22%)	397 (24%)	0.4
Ischemic heart disease, *n* (%)	208 (20%)	231 (14%)	<0.01
Congestive heart failure, *n* (%)	202 (19%)	152 (9%)	<0.01
Prior stroke, *n* (%)	208 (20%)	188 (11%)	<0.01
COPD, *n* (%)	126 (12%)	139 (8%)	<0.01
Dementia, *n* (%)	22 (2%)	9 (0.5%)	<0.01
Chronic kidney disease, *n* (%)	8 (1%)	93 (6%)	<0.01
Hemodialysis, *n* (%)	5 (0.5%)	66 (4%)	<0.01
Chronic liver disease, *n* (%)	46 (4%)	137 (8%)	<0.01
Solid tumor, *n* (%)	201 (19%)	420 (25%)	<0.01
Leukemia, *n* (%)	20 (2%)	106 (6%)	<0.01
Lymphoma, *n* (%)	16 (1.5%)	48 (3%)	<0.01
Solid organ transplantation, *n* (%)	3 (0.3%)	184 (11%)	<0.01
Lung transplant	0 (0%)	13 (1%)	<0.01
Heart transplant	0 (0%)	1 (0.06%)	1
Liver transplant	0 (0%)	43 (3%)	<0.01
Kidney transplant	3 (0.3%)	132 (8%)	<0.01
HST, *n* (%)	0 (0%)	32 (2%)	<0.01
Chronic steroids, *n* (%)	111 (11%)	360 (21%)	<0.01

CCI—Charlson comorbidity index; IQR—interquartile range; BMI—body mass index; ICU—intensive care unit; COPD—chronic obstructive pulmonary disease; HST—hematopoietic stem cell transplant.

**Table 2:** *E. coli* bacteremia characteristics and outcomes according to age.

Variable	≥80 Years (*n* = 1042)	<80 Years (*n* = 1675)	*p* Value
*E. coli* resistance type			
3rd GC susceptible	709 (68%)	1228 (73%)	<0.01
3rd GC resistant	330 (32%)	437 (26%)	<0.01
CRE	3 (0.3%)	10 (0.6%)	<0.01
Time to antibiotics (days)	0.40 (0.22–0.58)	0.37 (0.21–0.57)	0.04
Empiric antibiotics within 24 h	947 (91%)	1471 (88%)	<0.01
Appropriate empiric antibiotics	691 (66%)	1163 (69%)	<0.01
Central line at sepsis presentation	49 (4.7%)	256 (15%)	<0.01
Bacteremia source, *n* (%)			
Urine	632 (61%)	850 (51%)	<0.01
Hepatobiliary	184 (18%)	303 (18%)	
Primary	127 (12%)	303 (18%)	
Intra-abdominal	74 (7%)	121 (7%)	
Respiratory	15 (1%)	16 (1%)	
Wounds	9 (1%)	53 (3%)	
Venous catheters	1 (0.1%)	29 (2%)	
SOFA score, median (IQR)	4.50 (3–7)	4 (2–6)	<0.01
Mortality			
Death in 7 days	99 (10%)	122 (7%)	0.04
Death in 14 days	159 (15%)	178 (11%)	<0.01
Death in 30 days	232 (22%)	278 (17%)	<0.01
Admission days	8 (5–12)	8 (5–15)	0.03
Albumen level, median (IQR)	3.1 (2.8–3.5)	3.2 (2.8–3.6)	<0.01
Albumin level < 3 mg/dL	261 (25%)	326 (19%)	0.2
Creatinine level, median (IQR)	1.28 (0.93–1.92)	1.14 (0.78–1.86)	<0.01
White blood cell count, median (IQR)	11.4 (8.1–16.7)	9.7 (6.5–14.7)	<0.01

CRE—carbapenem resistance Enterobacteriaceae; SOFA score—Sequential Organ Failure Assessment score; IQR—interquartile range.

**Table 3:** Appropriateness of empiric antibiotics among hospitalized patients >80 years with *E. coli* bacteremia (*n* = 947).

Variable	Inappropriate Therapy (*n* = 256)	Appropriate Therapy (*n* = 691)	*p* Value	*p* Value (Corrected FDR)
Age, median (IQR)	87 (83–91)	87 (83–90)	<0.01	0.02
Female gender, *n* (%)	149 (58%)	427 (62%)	0.5	0.7
BMI, median (IQR)	26 (23–29)	26 (23–30)	0.9	1
Age adjusted CCI, median (IQR)	5 (4–8)	5 (4–8)	0.6	1
SOFA score, median (IQR)	5 (3–7)	4 (3–6)	<0.01	<0.01
Home residency, *n* (%)	135 (53%)	437 (63%)	<0.01	<0.01
Prior hospitalization in 90 days, *n* (%)	75 (29%)	196 (28%)	0.6	0.8
Prior surgery in 30 days	7 (3%)	9 (1%)	0.9	1
Days collect given, median (IQR)	0.39 (0.19- 0.55)	0.41 (0.22- 0.59)	<0.01	0.01
Mechanical ventilation, *n* (%)	24 (9%)	32 (5%)	0.01	0.04
Admission unit, *n* (%)				
Internal medicine	197 (77%)	632 (91%)	<0.01	<0.01
Surgery	58 (23%)	57 (8%)		
ICU	0 (0%)	1 (0.1%)		
Comorbidities				
Diabetes mellitus, *n* (%)	53 (21%)	152 (22%)	1	1
Ischemic heart disease, *n* (%)	53 (21%)	133 (19%)	0.1	0.3
Congestive heart failure, *n* (%)	56 (22%)	121 (18%)	0.1	0.3
Prior stroke, *n* (%)	51 (20%)	137 (20%)	1	1
Dementia, *n* (%)	5 (2%)	15 (2%)	0.8	1
COPD, *n* (%)	31 (12%)	84 (12%)	0.6	0.8
Chronic liver disease, *n* (%)	10 (4%)	35 (5%)	1	1
Chronic kidney disease, *n* (%)	2 (1%)	5 (1%)	0.05	0.1
Hemodialysis, *n* (%)	2 (0.8%)	3 (0.4%)	0.5	0.8
Solid tumor, *n* (%)	51 (20%)	125 (18%)	1	1
Leukemia, *n* (%)	8 (3%)	10 (1%)	0.6	0.8
Lymphoma, *n* (%)	1 (0.4%)	10 (1%)	0.03	0.9
Solid organ transplantation, *n* (%)	0 (0%)	2 (0.3%)	0.1	0.3
HCT, *n* (%)	0 (0%)	0 (0%)	0.6	0.8
Chronic steroids, *n* (%)	20 (8%)	78 (11%)	<0.01	0.01
Central venous line, *n* (%)	16 (6%)	27 (4%)	0.6	0.8
Bacteremia source, *n* (%)				
Urinary	126 (49%)	450 (65%)	<0.01	<0.01
Hepatobiliary	61 (24%)	107 (15%)		
Intra-abdominal	32 (13%)	35 (5%)		
Primary	28 (11%)	87 (13%)		
Wound	4 (1.5%)	5 (0.7%)		
Respiratory	5 (2%)	7 (1%)		
Mortality, *n* (%)				
Death in 7 days	34 (13%)	46 (7%)	<0.001	<0.01
Death in 14 days	50 (20%)	87 (13%)	<0.01	<0.01
Death in 30 days	71 (28%)	128 (19%)	<0.01	<0.01
Admission days, median (IQR)	9 (5–13)	8 (5–12)	<0.01	<0.01
E. coli resistance type, *n* (%)				
3rd GC susceptible *E. coli*	115 (45%)	529 (77%)	<0.01	<0.01
3rd GC resistant *E. coli*	139 (54%)	161 (23%)		
CRE	2 (0.8%)	1 (0.1%)		
Albumin level, median (IQR)	3.1 (2.8–3.4)	3.1 (2.8–3.5)	0.06	0.2
Albumin level < 3 mg/dL	67 (26%)	170 (25%)	0.5	0.8
Creatinine level, median (IQR)	1.35 (0.95–2.03)	1.25 (0.91–1.84)	0.9	1
White blood cell count, median (IQR)	12.7 (9.3–17.8)	10.9 (7.7–15.9)	<0.01	<0.01

BMI—body mass index; COPD—chronic obstructive pulmonary disease; ICU—intensive heart unit; HCT—hematopoietic stem cell transplant; IQR—interquartile range; 3rd GC—3rd generation cephalosporins; CRE—carbapenem resistance Enterobacteriaceae; SOFA score—Sequential Organ Failure Assessment score.

**Table 4:** Empiric antibiotic types administered to octogenarians in the appropriate empiric treatment group.

Antibiotic Type	3rd GC Susceptible *E. coli* (*n* = 529)	3rd GC Resistant *E. coli* (*n* = 161)	CRE (*n* = 1)
Cephalosporin (51.6%)	357 (67%)	-	-
Amikacin (33.7%)	119 (22%)	113 (71%)	1 (100%)
Piperacillin-tazobactam (6.2%)	24 (4.5%)	19 (11.8%)	-
Carbapenems (5.1%)	7 (1.3%)	28 (17.3%)	-
Quinolones (3.2%)	22 (4.1)	-	-
Others * (0.14%)	-	1 (0.6%)	-

* Others—Colistin. 3rd GC—3rd generation cephalosporin; CRE—carbapenem resistance Enterobacteriaceae.

**Table 5:** Univariate analysis of risk factors for 30-day mortality among hospitalized patients ≥80 years with *E. coli* bacteremia.

Variable	Survived (*n* = 810)	Dead (*n* = 232)	OR (CI95%)	*p* Value
Age, median (IQR)	86 (83–90)	88 (84–92)	1.04 (1.01–1.07)	<0.01
Female gender, *n* (%)	505 (62%)	124 (53%)	1.4 (1.1–1.9)	0.01
BMI, median(IQR)	26 (23–29)	25 (22–27)	0.92 (0.89–0.96)	<0.01
Age adjusted CCI, median(IQR)	5 (4–8)	6 (4–9)	1.06 (1.02–1.1)	<0.01
SOFA score, median (IQR)	4 (2–6)	7 (5–10)	1.4 (1.3–1.5)	<0.01
Home residency, *n* (%)	515 (64%)	120 (52%)	2 (1.5–2.8)	<0.01
Prior hospitalization in 90 days, *n* (%)	213 (26%)	93 (40%)	1.87 (1.38–2.54)	<0.01
Prior surgery in 30 days, *n* (%)	14 (1.7%)	4 (1.7%)	1.08 (0.4–3.2)	0.8
Admission unit, *n* (%)				
Internal medicine	703 (87%)	211 (91%)	1.5 (0.9–2.4)	0.3
Surgery	102 (13%)	21 (9%)		
ICU	3 (0.4%)	0 (0%)		
Mechanical ventilation, *n* (%)	30 (3.7%)	35 (15%)	4.6 (2.7–7.6)	<0.01
Empiric antibiotics within 24 h, *n* (%)	748 (92.35)	199 (86%)	0.5 (0.3–0.7)	<0.01
Days collect given, *n* (%)	0.41 (0.22–0.58)	0.37 (0.21–0.55)	0.9 (0.9–1.1)	0.9
Appropriate empiric, *n* (%)	563 (70%)	128 (55%)	0.59 (0.42–0.82)	<0.01
Comorbidities				
Ischemic heart disease, *n* (%)	152 (19%)	56 (27%)	1.38 (0.97–1.95)	0.07
Diabetes mellitus, *n* (%)	166 (20%)	66 (28%)	1.54 (1.1–2.1)	0.01
Prior stroke, *n* (%)	155 (19%)	53 (23%)	1.25 (0.88–1.78)	0.2
Congestive heart failure, *n* (%)	137 (17%)	65 (28%)	1.9 (1.36–2.69)	<0.01
COPD, *n* (%)	88 (11%)	38 (16%)	1.61 (1.1–2.4)	0.02
Solid tumor, *n* (%)	149 (18%)	52 (22%)	1.28 (0.9–1.8)	0.2
Chronic kidney disease, *n* (%)	8 (1%)	0 (0%)	0.2 (0.01–4.2)	0.3
Hemodialysis, *n* (%)	5 (1%)	0 (0%)	0.31 (0.01–7.5)	0.5
Solid organ transplant, *n* (%)	2 (0.25%)	1 (0.5%)	2.1 (0.2–21.7)	0.5
Chronic steroids, *n* (%)	86 (11%)	25 (11%)	1.3 (0.64–1.65)	0.9
Central venous line, *n* (%)	26 (3%)	23 (10%)	0.28 (0.16–0.5)	<0.01
Bacteremia source				
Urinary, *n* (%)	510 (63%)	122 (53%)	0.65 (0.48–0.87)	0.02
Hepatobiliary, *n* (%)	143 (18%)	41 (18%)		
Primary, *n* (%)	86 (11%)	41 (18%)		
Intra-abdominal, *n* (%)	55 (7%)	19 (8%)		
Respiratory, *n* (%)	9 (1%)	6 (3%)		
Infected wound, *n* (%)	6 (1%)	3 (1%)		
Admission days, median (IQR)	8(5–12)	8(4–13)	0.99 (0.97–1.01)	0.3
*E. coli* resistance type, *n* (%)				
3rd GC susceptible *E. coli*	586 (72%)	123 (53%)	0.43 (0.32–0.58)	<0.01
3rd GC resistant *E. coli*	223 (28%)	107 (46%)		
CRE	1 (0.12%)	2 (0.86%)		
Albumin level, median (IQR)	3.2 (2.9–3.5)	2.8 (2.3–3.2)	0.17 (0.11–0.25)	<0.01
Albumin level < 3 mg/dL	165 (20%)	96 (41%)	3.6 (2.5–5.2)	<0.01
Creatinine level, median (IQR)	1.2 (0.9–1.7)	1.7 (1.1–2.4)	1.33 (1.16–1.52)	<0.01
White blood cell count, median (IQR)	11.1 (8.1–15.7)	12.6 (8.4–20.2)	1.03 (1.01–1.05)	<0.01

BMI—body mass index; CRE—carbapenem resistance Enterobacteriaceae; 3rd GC—3rd generation cephalosporin; ICU—intensive care unit; IQR—interquartile range; SOFA score—Sequential Organ Failure Assessment score.

**Table 6:** Multivariate binary logistic regression model for risk factors of 30-day mortality in hospitalized patients > 80 years with *E. coli* bacteremia.

Variable	Adjusted Odds Ratio	*p* Value
Appropriate vs. inappropriate empiric therapy	1.10 (0.64–1.81)	0.7
SOFA score < 5 vs. >5 points	0.17 (0.010–0.31)	<0.01
Urinary vs other sources of bacteremia	0.71 (0.44–1.15)	0.2
3rd GC susceptible vs. resistant *E. coli*	0.43 (0.26–0.0.69)	0.9
Albumin level < 3 vs. >3 mg/dL	2.49 (1.56–3.97)	<0.01

3rd GC—3rd generation cephalosporin; SOFA score—Sequential Organ Failure Assessment score.

## Data Availability

The original contributions presented in this study are included in the article. Further inquiries can be directed to the corresponding author.
